# Regional Inequality in the distribution of dentists in Saudi Arabia by sector and nationality: A 2023 National analysis under vision 2030

**DOI:** 10.1371/journal.pone.0348204

**Published:** 2026-04-29

**Authors:** Waleed Kattan

**Affiliations:** Department of Health Services and Hospitals Administration, Faculty of Economics and Administration, King Abdulaziz University, Jeddah, Saudi Arabia; UCLA School of Dentistry, UNITED STATES OF AMERICA

## Abstract

**Objective:**

This study examines the distribution of dentists across Saudi Arabia’s regions in 2023, comparing the public and private sectors and Saudi- and non-Saudi dentists, to inform effective oral healthcare policies aligned with Vision 2030.

**Methods:**

Using data from the 2023 MOH Statistical Yearbook, dentist per 10,000 people ratios were calculated across 20 health regions. Regional disparities were assessed using the Gini coefficient and Lorenz curves, which evaluated inequalities by sector (MOH versus private sector) and nationality (Saudi versus non-Saudi).

**Results:**

Between 2019 and 2023, Saudi Arabia’s dental workforce experienced significant growth, reaching a density of 7.7 dentists per 10,000 people. However, regional disparities remain pronounced, with higher densities in smaller regions, such as Al-Bahah (9.0), compared to densely populated areas like Makkah (4.5). The private sector dominates dentist employment, accounting for approximately 70% of dentists, particularly in urban areas. It also exhibits significant regional distribution inequality, as indicated by a Gini coefficient of 0.60. Notably, the MOH relies heavily on non-Saudis (92%), which contrasts sharply with private-sector employment patterns, where 67% of dentists are Saudi.

**Conclusion:**

Despite overall improvements in dentist density, substantial regional and sectoral disparities persist. Effective policymaking requires targeted interventions that address geographic inequalities, incentivize public sector employment, and promote Saudization to achieve Vision 2030’s goal of equitable healthcare access nationwide. These results highlight persistent inequalities in the distribution of oral healthcare. Addressing these gaps through targeted regional planning, enhanced Saudization, and balanced public–private strategies will be crucial for realizing Vision 2030’s goals of equitable access.

## Introduction

Access to oral healthcare is crucial for maintaining good oral and overall health [1]. The geographical distribution of dental professionals directly impacts people’s ability to access dental services in their local communities [2,3]. Understanding the distribution of dentists across regions in Saudi Arabia is essential to ensuring equitable access to dental care nationwide [4]. Oral health is intrinsically linked to general health and quality of life. Lack of access to oral healthcare can negatively influence oral health status and disease management [5]. Timely treatment of dental issues prevents more severe, costly problems down the line [6]. Beyond individual health impacts, the inequitable distribution of dental professionals exacerbates oral health inequalities across communities [7]. Accurately assessing dentist requirements and distribution patterns across regions remains crucial for the effective development of long-term oral healthcare and informed policy decisions.

**Fig 1:**
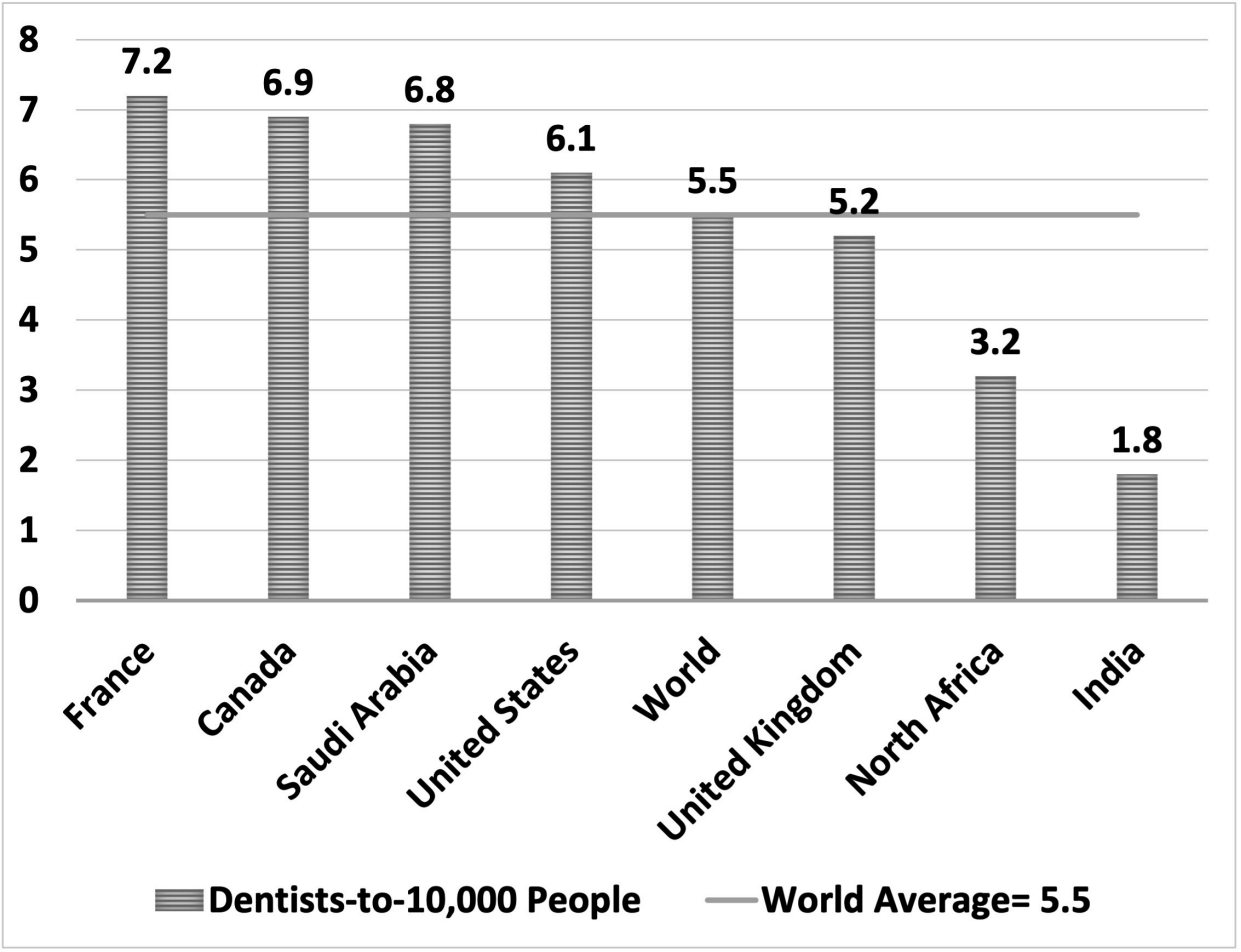
Contextual comparison of dentist density across selected countries in 2022 [8].

According to United Nations statistics for 2020, Saudi Arabia had 6.8 dentists per 10,000 people, higher than the global average of 5.5 that year [8] (Fig 1). However, these national figures provide an incomplete picture without examining intra-country variations. Local assessments of dentist numbers in specific regions are necessary to understand real geographic access to oral healthcare services. [9–12].

The Kingdom of Saudi Arabia has made significant progress in healthcare development since establishing its first public health department in 1925. Despite having a healthcare system ranked 26th globally by the World Health Organization (WHO), Saudi Arabia faces challenges in matching its rapid population growth with adequate healthcare human resources [13]. The Saudi Vision 2030 strategic plan acknowledges these challenges and emphasizes the need for a robust reserve of healthcare professionals, including dentists, to serve a population projected to reach 39.8 million by 2025 [13].

The vision is a strategic framework guiding the Kingdom’s development, with a growing emphasis on healthcare services, including dental care [14]. It emphasizes diversifying the economy and improving all public services through technology solutions. As part of this direction, efforts are being made to align the health sector with international standards and optimize service distribution and quality, including dental care [15]. The healthcare landscape in Saudi Arabia has undergone significant reforms in recent years, centered on more equitable geographic access to medical interventions across the country [16–18].

**Fig 2:**
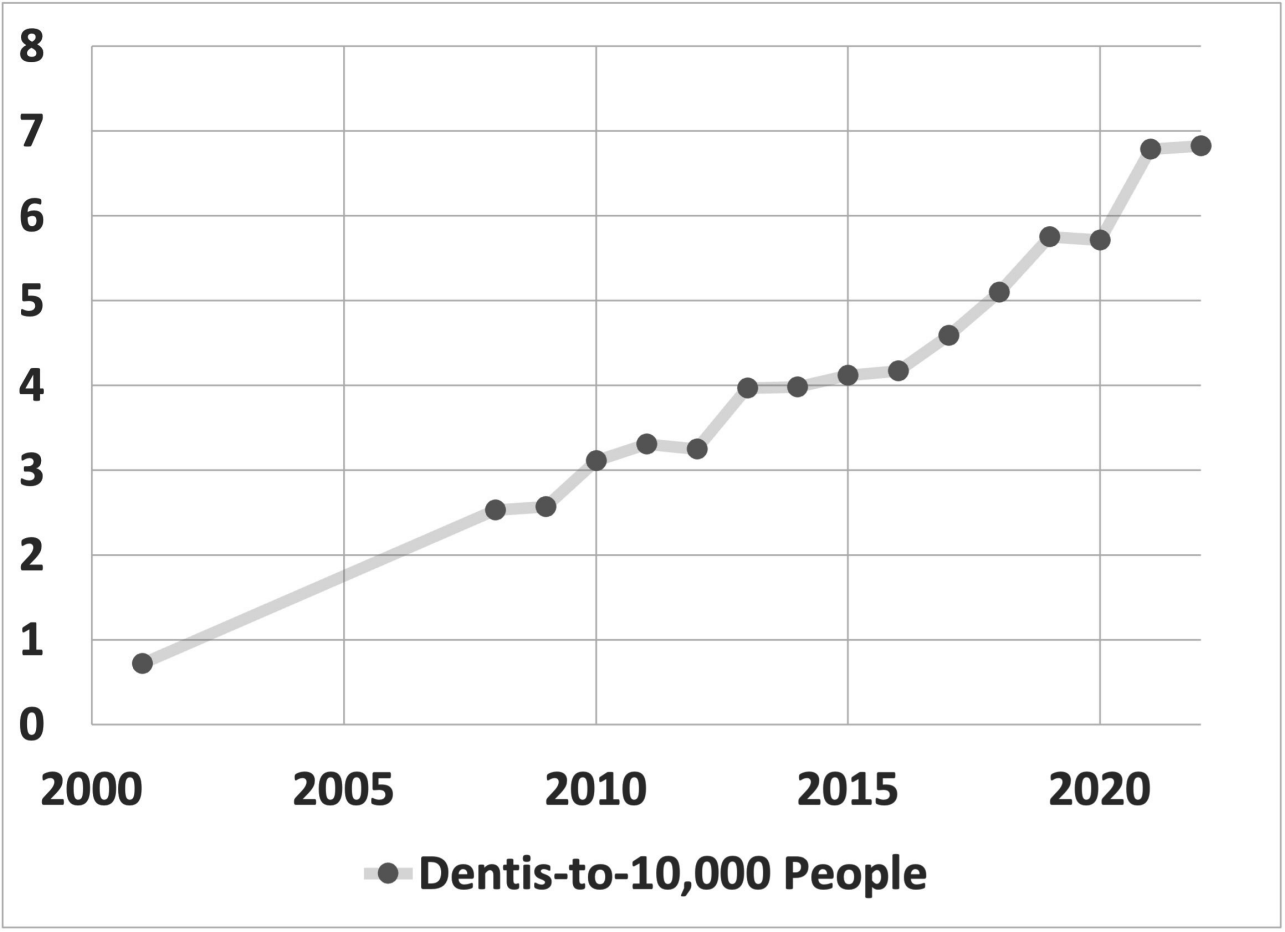
Historical trend in Saudi dentist density, 2001–2021 [20].

Alongside these national transformations, the dental workforce in Saudi Arabia has shown steady growth over the last 20 years [19]. Over the past two decades, the progressive increase in dentist density in Saudi Arabia has reflected significant advancements in the country’s healthcare infrastructure and a focused investment in dental health services. The Ministry of Health’s (MOH) efforts to bolster the number of healthcare professionals, including dentists, are evident in the nearly tenfold increase from 2001 to 2021 (Fig 2).

A key focus of reforms under Vision 2030 is introducing a new Model of Care, aiming to facilitate more equitable access to healthcare services nationwide, including dental services [15]. The proper distribution of health professionals is a recognized challenge globally and locally, profoundly impacting both access to care and the quality of services received [21]. Despite overall workforce increases, shortages may persist in some regions due to a lack of detailed empirical studies on geographic distribution patterns.

This study aims to provide an updated, region-specific analysis of the dentist workforce across Saudi Arabia in 2023, using the most recent data available under the Vision 2030 framework. By calculating dentist-to-population ratios for each region and comparing distributions by sector and nationality, the research identifies regional disparities that influence access to oral healthcare. Building upon earlier studies that relied mainly on national averages or older datasets [4,10,22], this analysis incorporates inequality metrics, such as the Gini coefficient and Lorenz curves, to provide a more detailed and contemporary assessment. The findings are intended to guide evidence-based workforce planning and policy development to promote equitable and sustainable dental care provision throughout the Kingdom. Accordingly, this study aims to analyze the regional distribution of dentists in Saudi Arabia in 2023, comparing the public and private sectors and Saudi and non-Saudi dentists, to inform effective oral healthcare planning and policymaking under Vision 2030.

### Regional disparities in dental care access

The geographical distribution of dentists is a critical factor influencing access to oral healthcare services [2]. Analysis of distribution patterns can identify disparities that may hamper equal access to dental care. In Saudi Arabia, several studies have highlighted disparities in dental service provision across regions [2,12,22,23]. Understanding these disparities and their underlying dynamics is crucial for informed nationwide policymaking regarding equitable oral healthcare delivery.

The dental workforce in Saudi Arabia has expanded significantly over the past few decades, primarily due to the growth of educational institutions and their capacity. However, the actual distribution of dentists does not necessarily reflect this quantitative growth [24]. In 2014, Abdelhay-Altamimi and Nawaf’s study provided valuable insights into workforce dynamics, revealing that only 22.08% of dentists in KSA were Saudi nationals, with the majority being non-Saudi [4]. Recent efforts have been made to increase the percentage of Saudi dentists; however, despite these efforts, approximately half of the dentists working in Saudi Arabia in 2023 were non-Saudis (Table 1).

**Table 1:** Dentists’ numbers and densities 2019-2023.

Year	Population	Total Dentists	Dentist per 10,000 People	Proportion of Saudi Dentists
**2019**	30,063,799	18,813	6.3	40%
**2020**	31,552,510	19,622	6.2	50%
**2021**	30,784,383	22,739	7.4	53%
**2022**	32,175,224	23,897	7.4	54%
**2023**	33,764,022	25,970	7.7	54%

The steady rise in dentist density, from 6.3 to 7.7 per 10,000 people, reflects both workforce expansion and increasing Saudization, the national policy promoting the employment of Saudi nationals across professional sectors. Yet nearly half of dentists remain non-Saudis, underscoring the continued reliance on expatriates.

This dependency on foreign dentists affects strategic workforce planning as the proportion of Saudi dentists rises. It also has implications for regional distribution. AlBaker et al.‘s 2017 study reported that over 80% of Saudi dentists worked in major regions, including Riyadh, Makkah, and the Eastern Province [4]. This suggests potential imbalances across regions not fully captured by national ratios alone. A more recent 2021 study by Shubayr, Kruger, and Tennant, focusing on the Jazan region, similarly found variations in dental facility distribution across cities [10]. These findings emphasize the need for region-specific analyses to validate overall disparities.

Access to dental facilities is concentrated in urban centers. A 2023 review highlighted healthcare challenges in Saudi Arabia, including inequitable access between regions, which could impact dental distribution [15]. Studies consistently report higher dentist densities in urban areas compared to rural areas [25]. The 2022 review by Alqahtani et al. specifically noted this maldistribution of dental professionals between urban and rural regions. [22]

Regional disparities are magnified in more remote areas. One 2022 systematic review of caries prevalence found considerable variability among Saudi regions, necessitating locale-tailored policies and resources [26]. The 2021 study by Quadri et al. also found that only a small proportion of the 275 dental clinics in the Jazan region were more than 20 km from city centers.

These accessibility gaps have real healthcare impacts [27]. Analyzing Al Madina City specifically, Alsharif’s 2020 study demonstrated wide variation in dentist-to-population ratios across neighborhoods [24]. Similarly, a 2021 UK-focused study by Jo et al. found lower dentist-to-population ratios in rural areas compared to urban areas [28]. Both suggest that geographic access affects oral health outcomes.

Government initiatives aim to address these challenges. Vision 2030’s new Model of Care promotes equitable nationwide access, including dental care [15,29]. However, a 2017 study by Banjar et al. found negative correlations between population densities in Saudi Arabia and health facility ratios, making it difficult to achieve a balanced distribution [23]. Therefore, further research is necessary to optimize dental resource allocation in accordance with population needs. Understanding changing dynamics, such as the rise in Saudi dentist representation, will further inform strategic workforce planning.

In summary, studies consistently indicate that dental services are unevenly distributed between urban and rural populations in Saudi Arabia. Gaps in accessibility and outcomes between regions underscore the importance of ongoing research to characterize distribution disparities and fully support equitable healthcare policymaking nationwide. Such evidence can help assess progress toward Vision 2030 goals of improving access across the Kingdom.

## Materials and methods

Based on the 2023 MOH Statistical Yearbook data, dentist-to-population ratios were calculated for each of Saudi Arabia’s 20 health regions, using the total number of dentists and the corresponding regional population sizes as of 2023 [20]. Specifically, the number of dentists in each region was divided by the population size of that region, and this value was multiplied by 10,000 to derive a standardized ratio of dentists per 10,000 people. Both the workforce and population figures were obtained from the 2023 MOH Statistical Yearbook, ensuring that each ratio reflects data from the same reference year. Although all data originated from the 2023 MOH Statistical Yearbook, the present analysis independently calculated region-specific dentist-to-population ratios, sectoral distributions, and inequality indices (Gini coefficients and Lorenz curves), providing new analytical insights beyond the raw administrative figures.

The Gini coefficient was employed to assess inequalities in the regional distribution of the dental workforce, calculated separately for two scenarios: firstly, comparing the distribution across employment sectors (total workforce vs. MOH vs. Private Sector [PS]), and secondly, comparing the distribution based on nationality (Saudi vs. non-Saudi dentists). The Gini coefficients were calculated using absolute counts of dentists across regions to reflect the actual distribution of the workforce and to measure regional inequality. Using absolute counts also allows direct comparison between sectors and workforce groups, independent of population size variations. The Gini coefficient was calculated using the standard cumulative proportion method commonly applied in health workforce inequality studies [30]. The Gini coefficient ranges from 0 to 1, where 0 indicates perfect equality, and 1 represents the most significant possible inequality [30]. Corresponding Lorenz curves were constructed to visually represent the distribution for both analyses, with greater deviations from the line of equality indicating increased disparities.

Dentists were classified by their main place of employment, as reported in the 2023 MOH Statistical Yearbook, which distinguishes between the MOH, the private sector, and other government sectors. Those listed under MOH facilities were considered MOH dentists, while those under private institutions were classified as private-sector dentists. Nationality (Saudi vs. non-Saudi) was based on the same source. Because the data reflect each dentist’s primary employer, those who might practice in multiple settings were counted once under their primary affiliation.

From the total 25,970 dentists reported in the 2023 MOH Statistical Yearbook, 83 dentists from MOH Headquarters and 2,238 from “Other Government Sectors” (such as the National Guard and Ministry of Defense hospitals) were excluded due to a lack of region-specific assignment. This yielded 23,649 dentists included in the final regional analysis.

## Results

### Workforce growth and dentist density (2019–2023)

Between 2019 and 2023, the dental workforce in Saudi Arabia grew overall, increasing from 18,813–25,970 dentists (Table 1). Although the trend was generally upward, minor fluctuations were observed, including a slight decline in density around 2020 and a near plateau between 2021 and 2022. Minor fluctuations were observed around 2020 and between 2021 and 2022. This overall growth was accompanied by a consistent rise in the proportion of Saudi dentists, from 40% in 2019 to 54% in 2023, indicating steady progress toward national Saudization goals. Despite these improvements, nearly half of all dentists remain non-Saudis, underscoring the continued reliance on expatriate professionals.

### Regional distribution and density of dentists (2023)

Descriptive statistics and inequality measures were used to summarize regional variations in dentist density and workforce composition. The 2023 regional analysis revealed significant variations in the distribution and density of dentists. Riyadh, Jeddah, and the Eastern region had the highest absolute numbers of dentists, with Riyadh alone accounting for approximately 29% of the country’s total dental workforce. Contrarily, dentist density was highest in less populous regions such as Al-Bahah (9.0 per 10,000 people) and Bishah (8.8 per 10,000 people), reflecting an imbalance between population size and dentist allocation. Meanwhile, densely populated regions like Jazan and Makkah exhibited notably lower dentist densities of 4.8 and 4.5 per 10,000 people, respectively, indicating regional variation in dentist density (Table 2).

**Table 2:** Distribution and density of dentists across regions in Saudi Arabia, 2023 *.

Region	Dentists	Proportion of Total Dentists	Population	Proportion of Total Population	Dentist per 10,000 People
**Al-Bahah**	320	1%	355,922	1%	9.0
**Bishah**	286	1%	324,812	1%	8.8
**Qaseem**	1,219	5%	1,402,158	4%	8.7
**Aseer**	1,512	6%	1,799,430	5%	8.4
**Jeddah**	3,282	14%	4,167,942	12%	7.9
**Riyadh**	6,810	29%	9,016,005	27%	7.6
**Ha’il**	586	2%	783,263	2%	7.5
**Najran**	452	2%	621,547	2%	7.3
**Qurayyat**	145	1%	204,645	1%	7.1
**Al-Jouf**	283	1%	420,597	1%	6.7
**Medinah**	1,496	6%	2,243,555	7%	6.7
**Hafr Al-Baten**	325	1%	490,067	1%	6.6
**Eastern**	2,467	10%	3,729,473	11%	6.6
**Al-Ahsa**	765	3%	1,158,795	3%	6.6
**Tabouk**	603	3%	929,788	3%	6.5
**Ta’if**	741	3%	1,155,966	3%	6.4
**Northern Borders**	238	1%	392,024	1%	6.1
**Qunfudah**	147	1%	283,619	1%	5.2
**Jazan**	711	3%	1,474,375	4%	4.8
**Makkah**	1,261	5%	2,810,039	8%	4.5
**Overall**	**23,649**	**100%**	**34,218,169**	**100%**	**(Average) 7.0**

*Table ranks regions by dentists-per-10,000-people.

The results in Table 1 demonstrate consistent workforce growth alongside notable structural shifts. Between 2019 and 2023, the number of dentists in Saudi Arabia rose from 18,813–25,970, increasing dentist density from 6.3 to 7.7 per 10,000 people. This trend reflects both population growth and sustained investment in dental education and training capacity. Importantly, the share of Saudi dentists rose from 40% to 54% over this period, signaling progress toward Saudization objectives and reduced reliance on foreign-trained professionals. Nevertheless, nearly 50% of the workforce remains non-Saudi, indicating continued reliance on expatriates, particularly in the public sector. The simultaneous rise in the number of dentists and Saudization indicates positive momentum, but the enduring reliance on non-Saudis highlights ongoing challenges in achieving workforce self-sufficiency.

### Sectoral distribution of dentists (2019–2023)

Between 2019 and 2023, sector-specific growth was particularly pronounced within the PS sector, with the number of dentists increasing to 16,676 by 2023, while the MOH employed only 7,056 dentists during the same year. This trend highlights the growing reliance on PS to meet dental care needs. Regional disparities were evident, particularly in Riyadh, where PS had a significant dominance of dentists (5,136) compared to those employed by the MOH (1,674). Conversely, regions such as Al-Bahah and Bishah exhibited more equitable distributions between MOH and PS, indicating regional variations in sectoral reliance (Tables 3 and 4).

**Table 3:** Distribution of dentists workforce across different health sectors in Saudi Arabia (2019-2023).

Year	Total dentists	Proportion of Saudis	MOH	PS	Other Gov.
**2019**	18,813	40%	4,843	12,285	1,685
**2020**	19,622	50%	5,815	12,039	1,768
**2021**	22,739	53%	6,810	13,931	1,998
**2022**	23,897	54%	7,153	14,891	1,853
**2023**	25,970	54%	7,056	16,676	2,238

**Table 4:** Distribution and density of dentists across regions in Saudi Arabia (MOH vs. PS) in 2023 *.

Health Region	MOH & PS		MOH		PS	
	Total Dentists	Dentists-to-10,000-People	MOH Dentists	Dentists-to-10,000-People	PS Dentists	Dentists-to-10,000-People
**Al-Bahah**	320	9.0	160	4.5	160	4.5
**Bishah**	286	8.8	151	4.6	135	4.2
**Qaseem**	1,219	8.7	489	3.5	730	5.2
**Aseer**	1,512	8.4	503	2.8	1,009	5.6
**Jeddah**	3,282	7.9	571	1.4	2,711	6.5
**Riyadh**	6,810	7.6	1,674	1.9	5,136	5.7
**Ha’il**	586	7.5	229	2.9	357	4.6
**Najran**	452	7.3	180	2.9	272	4.4
**Qurayyat**	145	7.1	68	3.3	77	3.8
**Al-Jouf**	283	6.7	148	3.5	135	3.2
**Medinah**	1,496	6.7	450	2.0	1,046	4.7
**Hafr Al-Baten**	325	6.6	137	2.8	188	3.8
**Eastern**	2,467	6.6	559	1.5	1,908	5.1
**Al-Ahsa**	765	6.6	257	2.2	508	4.4
**Tabouk**	603	6.5	227	2.4	376	4.0
**Ta’if**	741	6.4	211	1.8	530	4.6
**Northern Borders**	238	6.1	126	3.2	112	2.9
**Qunfudah**	147	5.2	71	2.5	76	2.7
**Jazan**	711	4.8	302	2.0	409	2.8
**Makkah**	1,261	4.5	460	1.6	801	2.9
**Total/ Average**	**23,649**	**7.0**	**6,973**	**2.0**	**16,676**	**4.9**

*Table ranks regions by MOH & PS dentists-per-10,000-people.

The results in Tables 3 and 4 confirm that workforce growth between 2019 and 2023 was driven primarily by the private sector, which expanded from 12,285–16,676 dentists, while the MOH remained relatively stable at around 7,000 dentists. By 2023, nearly 70% of dentists were employed in the private sector, compared to less than one-third in MOH. In Riyadh and Jeddah, private-to-MOH ratios exceeded 3:1 and 5:1, respectively, while smaller regions such as Al-Bahah and Bishah showed near parity. These disparities mirror dentist-to-population ratios, with smaller regions recording some of the highest values, while populous regions like Makkah and Jazan fell well below the national average. Private sector dentists were mainly concentrated in major regions such as Riyadh and Jeddah, while smaller regions such as Al-Bahah and Bishah showed more balanced distributions.

### Nationality distribution across sectors and regions (2023)

Regarding the distribution of nationalities in 2023, a striking disparity was observed between MOH and PS. PS demonstrated a higher reliance on Saudi dentists (average 67%) than MOH (average 8%), indicating a significant reliance on non-Saudi personnel by MOH. The highest proportion of Saudi dentists in PS was in Hafr Al-Baten and Bishah, each reaching 87%. The MOH had relatively higher proportions of Saudi dentists in regions such as the Northern Borders (29%) and Al-Jouf (28%). Still, the overall low percentages across most regions suggest a heavy reliance on non-Saudis within governmental dental services (Table 5).

**Table 5:** Regional trends in the percentages of Saudi Dentists (MOH vs. PS) in 2023 [20].

	MOH & PS			MOH			PS		
Health Region	Total	Total Saudis	Proportion of Saudis	Total	Total Saudis	Proportion of Saudis	Total	Total Saudis	Proportion of Saudis
Qunfudah	147	77	52%	71	14	20%	76	63	83%
Qurayyat	145	69	48%	68	15	22%	77	54	70%
Northern borders	238	135	57%	126	36	29%	112	99	88%
Bishah	286	142	50%	151	24	16%	135	118	87%
Al-Jouf	283	144	51%	148	42	28%	135	102	76%
Al-Bahah	320	154	48%	160	23	14%	160	131	82%
Hafr Al-Baten	325	178	55%	137	14	10%	188	164	87%
Najran	452	244	54%	180	26	14%	272	218	80%
Ha’il	586	298	51%	229	29	13%	357	269	75%
Tabouk	603	327	54%	227	23	10%	376	304	81%
Jazan	711	271	38%	302	15	5%	409	256	63%
Al-Ahsa	765	337	44%	257	17	7%	508	320	63%
Ta’if	741	458	62%	211	23	11%	530	435	82%
Qaseem	1,219	626	51%	489	103	21%	730	523	72%
Makkah	1,261	565	45%	460	24	5%	801	541	68%
Aseer	1,512	701	46%	503	18	4%	1,009	683	68%
Medinah	1,496	761	51%	450	26	6%	1,046	735	70%
Eastern	2,467	1,256	51%	559	11	2%	1,908	1,245	65%
Jeddah	3,282	1,650	50%	571	9	2%	2,711	1,641	61%
Riyadh	6,810	3,257	48%	1,674	36	2%	5,136	3,221	63%
Total/ Average	**23,649**	**11,650**	**49%**	**6,973**	**528**	**8%**	**16,676**	**11,122**	**67%**

*Table ranks regions by MOH & PS total dentists.

Table 5 shows that Saudization is concentrated in the private sector (with an average of 67%), but remains minimal in the MOH (8%). Private sector dominance is strongest in Hafr Al-Baten, Bishah, and the Northern Borders, where over 85% of dentists are Saudi. In contrast, MOH facilities in Riyadh and Jeddah employ only 2% Saudi dentists, reflecting near-total reliance on expatriates. These findings demonstrate that while Saudization has advanced substantially in the private sector, progress within the MOH remains limited. This raises concerns about the sustainability of governmental dental services and the need for stronger strategies to train and retain Saudi dentists in public practice.

### Inequality in dentist distribution (Gini coefficient analysis)

**Fig 3:**
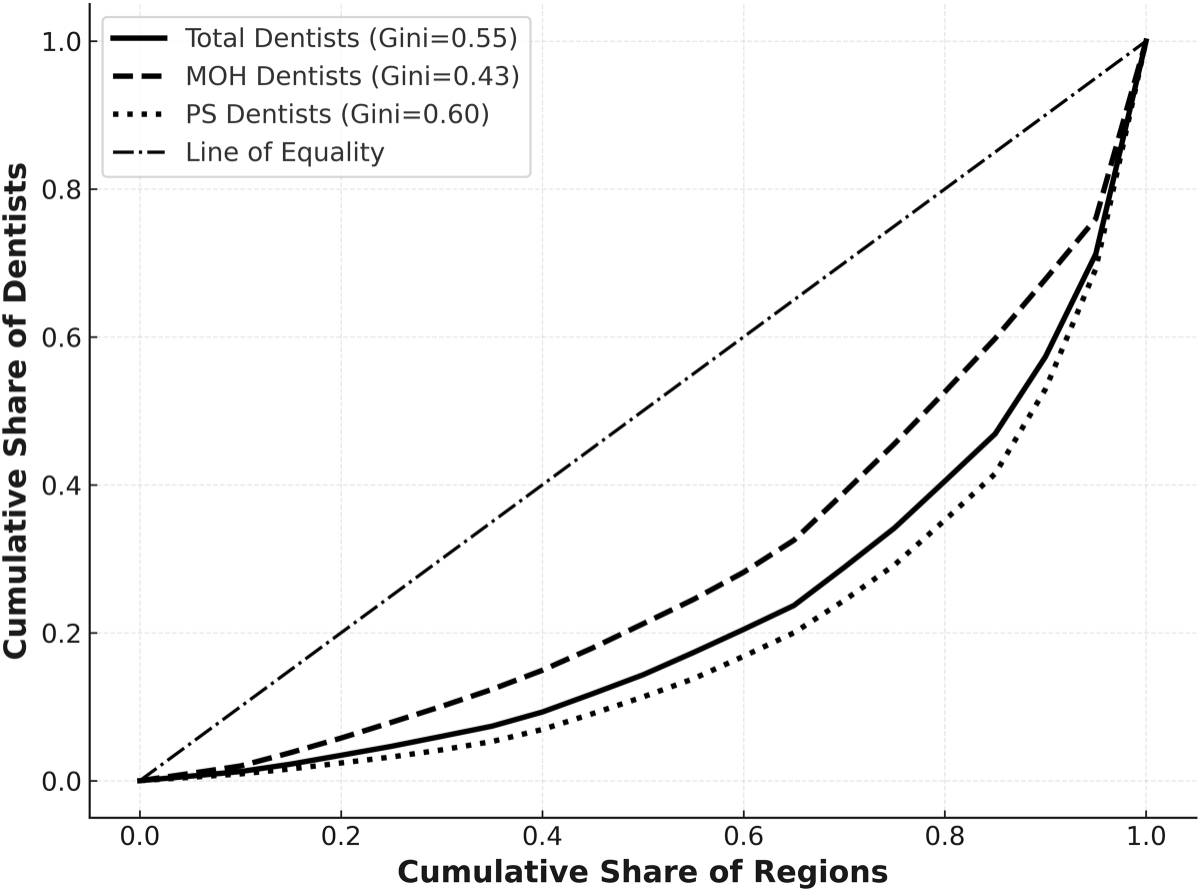
Inequality in dentist distribution by sector in Saudi Arabia, 2023.

The Gini coefficients for dentist distribution highlighted substantial disparities, particularly in PS, with a high value of 0.60, suggesting extreme regional disparities. Conversely, the MOH sector exhibited less inequality, with a Gini coefficient of 0.43, while the overall workforce showed moderate inequality at 0.55 (Fig 3).

**Fig 4:**
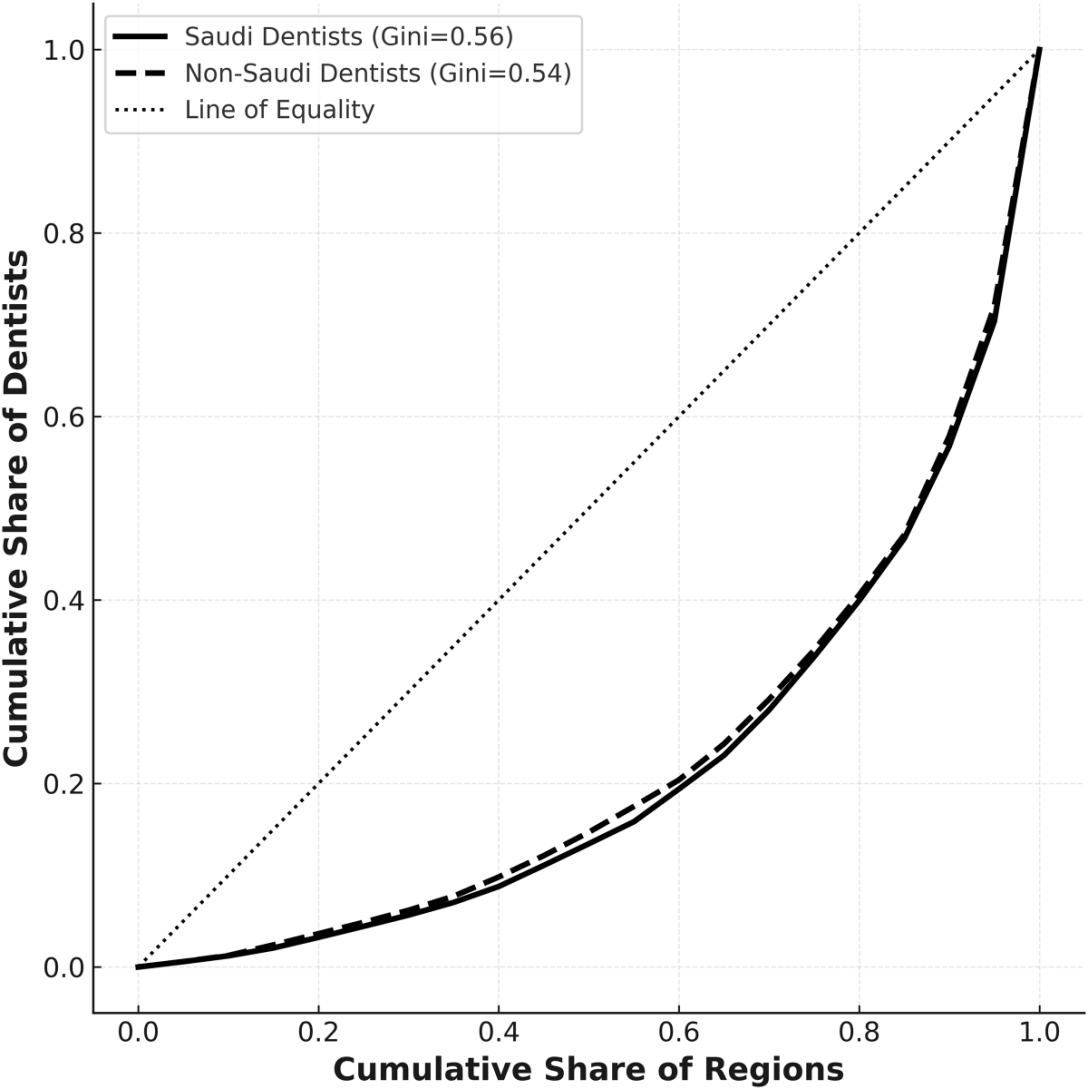
Geographic inequality in Saudi vs. Non-Saudi Dentists, 2023.

Regarding nationality-based distribution, both Saudi (0.56) and non-Saudi (0.54) dentists showed similar, moderately high levels of inequality, indicating consistent regional disparities across nationalities (Fig 4).

## Discussion

Although Saudi Arabia’s dentist density (7.7 per 10,000) exceeds the global average (5.5 per 10,000), this national figure masks significant regional disparities [8]. This international benchmark reflects 2022 data from Our World in Data; minor variations across datasets occur due to year and source definitions. In many OECD and high-income countries, dentists or dentistry personnel densities cluster in higher ranges (for example, dentistry personnel datasets from WHO/Our World in Data) [31,32]. The comparison suggests that while Saudi Arabia does well globally, its performance is roughly in line with that of high-income countries rather than dramatically ahead. This nuance is important because the national average may conceal significant internal imbalances. This overall performance reflects recent investments in health workforce expansion but conceals marked internal disparities among regions [19].

The analysis highlights crucial implications for healthcare policymakers, emphasizing the importance of addressing regional disparities in dental care distribution. The significant variance in dentist density across regions, particularly higher densities in less populous areas such as Al-Bahah and Bishah compared to densely populated areas such as Jazan and Makkah, suggests the need for tailored regional strategies. This necessitates targeted interventions to ensure equitable access to dental healthcare nationwide [11]. Such measures align with global health equity principles, emphasizing workforce distribution as a determinant of healthcare accessibility. Prior studies by Gallagher and Wilson [7] and Alshammari et al. [26] highlight the need for nuanced workforce planning, while Alsaeed et al. [33] and Alsalleeh et al. [34] demonstrated how patient preferences and the clustering of private clinics affect service access.

The substantial growth of dentists within the private sector compared to the MOH highlights the evolving role of private healthcare providers in Saudi Arabia’s dental care landscape. The results confirm the structural dominance of the private sector and the persistent regional disparities in access to dental care, with higher ratios often found in smaller regions and lower ratios in high-demand urban centers. This pattern is strongest in major regions such as Riyadh and Jeddah, where private institutions drive workforce growth. While this growth enhances service availability in urban centers, it risks deepening disparities in regions where private investment remains limited [11,33].

The concentration of private dental services in major urban centers such as Riyadh, Jeddah, and the Eastern Province can be partly attributed to economic and regulatory factors. Larger metropolitan areas offer greater demand, higher patient purchasing power, and more favorable business conditions for private practice. Licensing policies and market dynamics also tend to favor urban investment, where infrastructure, specialized suppliers, and professional networking opportunities are more accessible [34]. Additionally, studies have shown that patient preferences often lean toward private dental services in metropolitan areas due to shorter waiting times and perceived higher service quality [33]. These economic and behavioral factors together contribute to the spatial clustering of private dental clinics in high-income regions, leaving smaller provinces comparatively underserved.

Marked differences in nationality composition between sectors reveal another dimension of inequality. The private sector employs a higher proportion of Saudi dentists (67%). In contrast, MOH facilities remain heavily reliant on non-Saudis (8%), revealing uneven progress of Saudization, advancing rapidly in private practice but lagging in public services. Strengthening recruitment and retention programs for Saudi dentists in governmental institutions will be critical for achieving long-term workforce stability and alignment with Vision 2030 objectives [15].

Together, these sectoral and nationality-based patterns reveal structural disparities that affect both access and workforce sustainability. Policies aligning public and private contributions will be essential to achieving equitable and sustainable oral healthcare across all regions.

The Gini coefficient results confirm persistent regional disparities, especially within the private sector (Gini = 0.60), compared to the more balanced MOH system (Gini = 0.43). These findings indicate that unregulated market growth amplifies inequality, while coordinated planning can promote more balanced workforce distribution [7,26,35].

Reducing these disparities requires evidence-based policies beyond conventional recruitment incentives. Effective measures include loan-forgiveness or bonded-scholarship programs for dentists serving in underserved areas, housing and relocation allowances, and expanded postgraduate training in peripheral regions [22,36–38]. Public–private partnerships can also extend service coverage through government-supported rural clinics. These policy directions align with Vision 2030’s goal of equitable, self-sufficient healthcare [19,29,39]. Establishing a national monitoring framework to track dentist distribution and retention will support adaptive, data-driven planning.

## Limitations

The study’s reliance on secondary data from the 2023 MOH Statistical Yearbook presents several limitations. As an administrative dataset, the Yearbook may be subject to reporting inconsistencies or classification errors, particularly concerning regional allocations and sectoral categorization. Furthermore, the exclusive focus on 2023 does not account for subsequent changes in the healthcare landscape, including shifts in the balance between Saudi and foreign dentists, as well as the lingering effects of recent global health challenges. Additionally, since the Gini coefficient measures only a single point in time (2023), it cannot reflect changes in inequality over time. These factors may influence the current and future distribution of dental professionals, highlighting the need for ongoing research using updated data to maintain accuracy and relevance.

Additionally, this study excluded dentists employed by “other government sectors” and specialized governmental hospitals, as region-specific data were unavailable. This exclusion may introduce some bias, particularly in regions where these facilities constitute a notable share of healthcare services (e.g., Riyadh and the Eastern Region). Consequently, dentist densities in such areas may be slightly underestimated, and regional disparities could be somewhat overstated.

Further limitations include the potential underrecording of dentists practicing across multiple practice settings, as the Yearbook attributes each professional to a single primary employer. The study also did not incorporate measures of service need, utilization, or quality of care, which are necessary to contextualize workforce adequacy beyond numerical ratios. While dentist density provides a useful structural indicator, it does not capture differences in patient access, demand, or clinical quality across regions. These factors should be addressed in future studies to complement administrative workforce data with outcome- and need-based measures for a more comprehensive assessment of oral healthcare equity.

## Recommendations

To convert broad goals into actionable policy, Saudi Arabia might adopt loan forgiveness or debt-repayment schemes tied to mandatory service in underserved or rural regions [38]. Such approaches have proved effective in improving retention in challenging settings, particularly when combined with bonded scholarships or service-obligation contracts that require recipients to serve in high-need areas for a defined period. Moreover, aligning admissions policies to favor candidates from rural or underserved provinces and embedding rural clinical rotations during training can bolster local retention, a strategy supported by workforce development literature in the Eastern Mediterranean region [36].

Given Saudi Arabia’s expanding dental postgraduate training programs (which have significantly increased specialty seats between 2013 and 2023), it is critical to channel these programs to underserved provinces, offering incentives such as stipends, housing support, or accelerated promotion for those practicing in peripheral areas [22]. Complementing this, public–private partnership models should be encouraged, for instance, enabling private clinics to operate in rural zones under government subsidies or guaranteed service contracts. Finally, embedding a robust monitoring and evaluation framework that tracks longitudinal retention, turnover, utilization, and quality outcomes will help policymakers refine these interventions over time [37].

## Conclusion

This study provides an updated, region-specific assessment of Saudi Arabia’s dental workforce for 2023, revealing both substantial progress and persistent disparities. While the national dentist-to-population ratio now exceeds global averages, significant regional and sectoral imbalances remain, most notably, the concentration of private-sector dentists in major cities and the MOH’s continued reliance on non-Saudi professionals. These findings underscore the importance of targeted, evidence-based policies that promote equitable distribution, strengthen public-sector capacity, and advance Saudization goals. As this analysis is based on administrative Yearbook data, the absence of indicators related to service quality or patient need limits the ability to evaluate proper access or performance differences across regions. Under the Vision 2030 framework, addressing these imbalances through coordinated workforce planning, rural service incentives, and enhanced monitoring systems will be essential to achieving equitable access to quality dental care nationwide. Future studies should build on these findings by linking workforce distribution to patient outcomes and by tracking ongoing progress toward Saudization and service equity across Saudi regions.
